# Pancreatic Incidentaloma

**DOI:** 10.3390/jcm11164648

**Published:** 2022-08-09

**Authors:** Miłosz Caban, Ewa Małecka-Wojciesko

**Affiliations:** Department of Digestive Tract Diseases, Medical University of Lodz, 90-153 Lodz, Poland

**Keywords:** cysts, early detection, incidentaloma, neoplasm, pancreas, pancreatic cancer, pancreatic tumor

## Abstract

Pancreatic incidentalomas (PIs) represent a clinical entity increasingly recognized due to advances in and easier access to imaging techniques. By definition, PIs should be detected during abdominal imaging performed for indications other than a pancreatic disease. They range from small cysts to invasive cancer. The incidental diagnosis of pancreatic cancer can contribute to early diagnosis and treatment. On the other hand, inadequate management of PIs may result in overtreatment and unneeded morbidity. Therefore, there is a strong need to evaluate the nature and clinical features of individual PIs. In this review, we summarize the major characteristics related to PIs and present suggestions for their management.

## 1. Introduction

A pancreatic incidentaloma (PI) is defined as a lesion of the pancreas detected by imaging techniques performed for causes unrelated to the change itself or its implications. PIs may histologically correspond to a wide range of pathological conditions, from small cysts to large invading pancreatic tumors. The recent development of imaging techniques and their use contributed to the increase in the incidentaloma detection [1]. Incidental lesions are most often identified in organs, such as the thyroid gland, pituitary gland, kidney and lungs. In turn, the pancreas is characterized by lower spread of incidentalomas. However, PIs belong to the changes with higher rates of the potential malignancy compared to incidentalomas of other organs [2]. The random identification of PI may induce unnecessary anxiety in the patients and require useless diagnostics. In contrast, in cases of pre-malignant and malignant lesions, the detection of PI may be associated with the early diagnosis of treatable tumor and may prove to be lifesaving. Therefore, an assessment of PI is needed to implement adequate therapy, when medically indicated. Nevertheless, the discovery of PIs is a challenge for clinicians. It results from the fact that choice of appropriate investigations of PIs depends on a lot of factors, and management is not fully precise. Clinicians should consider numerous issues at diagnosis: how far its nature should be evaluated, lesion potential impact on the pancreas and adjacent structures, what follow-up is needed if at all, how the risk of missing a harmful diagnosis and a therapeutic opportunity against the risk of overdiagnosis can be balanced, as well as the patient stress. Hence, to gain a clearer understanding of the PIs and to help guide diagnosis and management, this review discusses the current state of knowledge on PIs, focusing on potential types of changes as PIs. We also emphasize the radiological and biochemical characteristics of individual PIs to facilitate proper, early diagnosis and pay attention to novel markers helpful for the diagnosis of PIs.

## 2. General Characteristics and Epidemiology

Initially, in 2009, the overall prevalence of PIs was estimated between 0.01–0.6% [3]. However, this rate may be considerably higher. An analysis of the pancreatic surgical resection series revealed that the frequency of asymptomatic neoplastic lesions is as high as 6–23% [4]. It is estimated that approximately 24 to 50% of them are malignant, and 24 to 47% are considered as potentially malignant or pre-malignant [5]. In turn, due to increasing abdominal imaging use and the general population aging, another study suggests that pancreatic cysts have been incidentally detected in up to 20% of patients in magnetic resonance imaging (MRI), and often even more in older subjects [6]. According to the most recent evidence, cystic lesions of the pancreas are widespread, often associated with incidental diagnosis, and their incidence may reach up to even 49% in the general population [7]. The vast majority of the detected pancreatic cysts are benign and only approximately 3% of them are malignant or potentially malignant [8].

**Figure 1:**
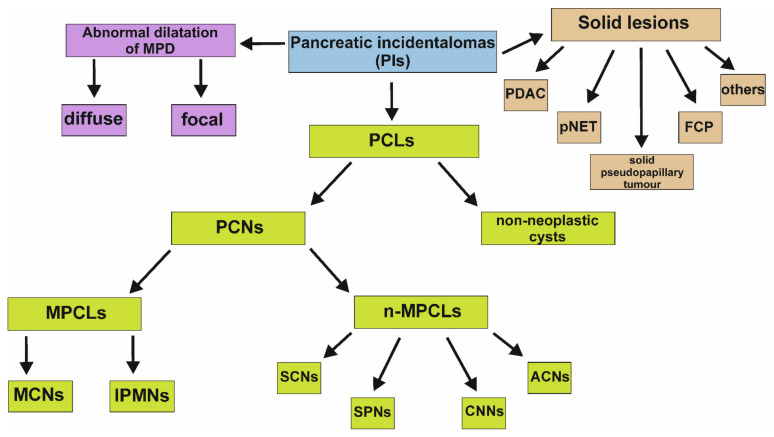
Subgroups of PIs. It is worth emphasizing that abnormal dilatation of the MPD may coexist with other types of PIs. ACNs, acinar-cell cystic neoplasms; CNNs, cystic neuroendocrine neoplasms; FCP, focal chronic pancreatitis; IPMNs, intraductal papillary mucinous neoplasms; MCNs, mucinous cystic neoplasms; MPCLs, mucinous pancreatic cystic lesions; n-MPCLs, non-mucinous pancreatic lesions; PCLs, pancreatic cystic lesions; PCNs, pancreatic cystic neoplasms; PDAC, pancreatic ductal adenocarcinoma; PIs, pancreatic incidentalomas; pNET, pancreatic neuroendocrine tumor; SCNs, serous cystic neoplasms; SPNs, solid-pseudopapillary neoplasms.

The etiology of PIs is multiple, complex, and depends on the nature of the lesion. Pathological changes may comprise benign adenoma, malignant adenocarcinoma or borderline malignant tumor with moderate dysplasia. In addition, PIs can include the following: mesenchymal tumors, endocrine tumors, cysts, congenital changes (among them choledochocele), intrapancreatic accessory spleen, metastatic lesions, infectious masses induced by *Ascaris lumbricoides*, *Candida albicans*, *Cytomegalovirus*, *Coxsackievirus*, *Cryptosporidium*, Mumps virus, *Mycobacterium* or others, non-islet tumors, and inflammatory masses [3]. Morphologically, there are three different types of PIs: solid lesions, cystic lesions, and abnormal dilatation of the main pancreatic duct. In addition, all of them may be divided into three following categories: benign, pre-malignant, and malignant lesion [2]. The pancreatic cysts, known also as pancreatic cystic lesions (PCLs), include both non-neoplastic and neoplastic cysts. The first of these comprise pseudocysts caused mainly by pancreatic inflammation or traumatic injury. Therefore, an identification of the cyst with no history of pancreatitis or injury in asymptomatic subjects in particular, suggests the suspicion of neoplastic cyst, also known as pancreatic cystic neoplasm (PCN). The classification of PCN is complex. There are two group of PCN: mucinous PCLs (MPCLs) and non-mucinous PCLs (n-MPCLs). MPCLs are divided into intraductal papillary mucinous neoplasms (IPMNs) and mucinous cystic neoplasms (MCNs), and n-MPCLs include serous cystic neoplasms (SCNs), solid pseudopapillary neoplasms (SPNs), cystic neuroendocrine neoplasms (CNNs), and acinar-cell cystic neoplasms (ACNs) [9] (Figure 1). IPMNs are the most representative group of PCN and constitute about 38% of PCLs. MCNs, SCNs and CNNs represent 23%, 16% and 7% of PCLs, respectively [10]. IPMNs are subdivided into main-duct IPMN, branch-duct IPMN and combined type of IPMN depending on an involvement of the pancreatic ductal system. Branch duct-IPMN is the most popular type of IPMN with the rate 46% of all IPMN, followed by combined type-IPMN (40%) and main duct-IPMN (14%) [9,11].

In turn, the percentage of solid pancreatic incidental lesions varies in retrospective studies, however, this is calculated to be between 31–65% of all PIs. The spectrum of detected solid PIs is broad and comprises malignant changes, such as exocrine, endocrine, lymphoproliferative, pancreatic ductal adenocarcinoma (PDAC) or metastases, pre-malignant lesions, primarily solid pseudopapillary tumor and pancreatic neuroendocrine tumor (pNET), foci occurring in chronic inflammation of the pancreas, and others (Figure 1). It is worth emphasizing that PDAC, pNET, solid pseudopapillary tumor and focal chronic pancreatitis are four most common solid incidental lesions with the incidence approximately ranging between 31–34%, 23–42%, 3–15%, 0–11% among solid PIs, respectively [12].

Abnormal dilatation of the main pancreatic duct (MPD) is associated with the various pancreatic diseases and may result from the existence of previously described solid or cystic lesions. However, it can also coexist with disorders of other organs, especially adjacent to the pancreas or occur in the patients with no pathology [13].

In the following chapter, we describe relevant features of pancreatic tumors, including their imaging, that may be of help in the differential diagnosis.

## 3. Types of Incidental Pancreatic Lesions

### 3.1. PCLs

Generally, PCLs are structures filled with fluid and may be found within or adjacent to the pancreas. In addition, some of them are characterized by a solid appearance, hindering proper diagnosis [14]. Higher incidence of PCLs is observed among elderly people, and is about 37% in adults aged over 80 [15]. Evaluation of pancreatic cysts should be performed in order to exclude or confirm its malignant nature. Various imaging methods are accessible to evaluate PCLs, including MRI, computed tomography (CT), and endoscopic ultrasound (EUS) with or without fine needle aspiration (FNA) [6,16]. Each of those techniques have advantages and disadvantages. For example, CT is widely available with short acquisition times, however, it is associated with exposure to ionizing radiation, as well as iodinated contrast media. MRI is characterized by high soft tissue contrast resolution and is also time-consuming. EUS may provide high spatial distribution imaging and enables FNA for an analysis of the PCLs fluid, but it is an invasive procedure [17]. The cystic fluid analysis is helpful in differentiating MPCLs from n-MCPLs. Nevertheless, it has limitations for distinguishing benign and malignant cystic lesions. Firstly, there are contradictory data on the cut-off value of markers, mainly carcino-embryonic antigen (CEA) and carcinoma antigen 19-9 (CA 19-9), differentiating benign and malignant lesions. Secondly, the molecular analysis of pancreatic cyst fluid, including genomic and epigenomic assay (genetic mutations, gene silencing, chromosomal deletions, heterozygosity loss) is still in progress [18,19]. Moreover, it was indicated that current imaging cannot accurately differentiate malignant from benign PCLs [20,21]. Despite the difficulties, there are some worrisome features suggesting the high risk of malignancy of PCLs, such as: the size of cyst (≥3 cm), dilatation of the main pancreatic duct (≥5 mm), the presence of a mural nodule, lymphadenopathy, thickened/enhancing walls of PCLs, sudden change in the format of the pancreatic duct concomitant with distal pancreatic atrophy, elevated serum level of CA 19-9, or rapid growth of lesion determined as ≥5 mm per two years rate [19,22,23].

#### 3.1.1. Non-Neoplastic Pancreatic Cysts

True cysts, most of which are inborn, and pseudocysts, primarily formed as a result of inflammation or injury, belong to the benign, non-neoplastic pancreatic cysts. In the past, about 80% of PCLs were considered to be pseudocysts. The current consensus indicates that only approximately 30% of PCLs are pseudocysts. This change in tendency results primarily from the development of EUS-FNA and its increased use [24,25,26]. Pseudocysts contain fluid collection surrounded by a wall of fibrous tissue without epithelium lining and are connected with the pancreatic duct system, either as a direct communication or indirectly through the pancreatic parenchyma. The diagnosis of a pseudocyst is related to a patient history compatible with pancreatitis, with additional evidence from laboratory and imaging features. However, clinicians should remember that patients with PCNs may also have had pancreatitis, and that subjects with a pseudocyst can have no apparent history suggestive of inflammation of the pancreas. For example, some extrapancreatic disorders, such as peptic ulcer disease, gastric cancer, ovarian cancer, abdominal aortic aneurysm, intestinal ischemia, bowel obstruction, myocardial infarction and pneumonia, may mimic the clinical presentation of pancreatic pseudocysts [27]. In addition, it seems that male sex predisposes patients to the pseudocyst, and its location in the pancreas is distributed evenly (no pancreatic region typical for pseudocyst) [27]. The endosonographers developed the ”string sign” test, helpful in differentiating mucin containing cyst from other cystic lesions. In this test, an operator places a sample of the aspirated fluid from the PCL between the thumb and index finger and separates the fingers in order to measure the distance between the fingers before the string breaks. The formation of a long string due to a high concentration of mucus indicates mucinous cysts. Elevated levels of amylase and lipase in the cyst fluid with negative string sign and cystic fluid CEA concentration <5 ng/mL are characteristic for pseudocysts [28]. Interestingly, the level of CA 19-9 in the fluid may be variable [27]. Moreover, they are often morphologically uniocular in ultrasound that may also demonstrate the echoic structure associated with distal acoustic enhancement [24]. On the initial stages of the lesion development, pseudocysts appear more complex, which may result from the presence of necrotic debris, immediately after acute necrotizing pancreatitis, in particular. Nonetheless, the identification of a thick-walled, rounded, fluid-filled lesion adjacent to the pancreas on an abdominal CT scan in a patient with a history of pancreatitis is typical for the pseudocyst. In this clinical situation, CT findings do not require confirmation with another diagnostic modality [27].

On the other hand, true cysts include congenital, duplication and retention cysts and are lined by epithelium. They represent less than 1% of all PCLs, and their differential diagnosis is difficult. Congenital cysts are most predominately found in childhood, probably caused by a developmental anomaly of the pancreatic ductal system. Their most common locations are the neck and tail of the pancreas, but they also may occur in other parts of the gland. Typically, they have a diameter smaller than 2–3 cm and may be solitary or multiple. The level of amylase in congenital pancreatic cysts may be higher compared to the serum levels, but is still lower than in retention cyst developed from the pancreatic duct dilatation. Generally, the amylase concentration of the congenital cystic fluid is approximately 300 U/l, while in the retention cyst, it ranges from 1000–3000 U/l [29].

#### 3.1.2. MPCLs

As demonstrated on the Figure 1, MPCLs include both IPMNs and MCNs. IPMNs are lesions of variable invasiveness, they may have benign, borderline, low-grade-dysplasia or invasive character. The changes derived from the epithelium of the pancreatic duct system are benign, and the tumor cells are tall, columnar, mucous-rich epithelial cells, widely invading the MPD and/or branch pancreatic duct (BPD) [30]. For IPMNs, the malignancy rate differs depending on the morphologic type. The risk rate of malignancy is the lowest for IPMN involving the branch ducts, and even between 33–60% when MPD is involved alone or in combination with BPD [31]. On the other hand, according to the histological evaluation and mucin expression, IPMNs may be divided into four epithelial subtypes: gastric-, oncocytic-, intestinal-, pancreatobiliary-type, and each of them have various type of the malignant progression risk. The first two are often low-grade neoplasms, while the intestinal- and pancreatobiliary-type of IPMNs have a tendency to be of high risk and are usually associated with invasive cancer disease [32]. Pancreatic cancer disease prevalence in IPMN is estimated to be between 6% up to as high as 46% [8,33,34]. In addition, the prognosis of IPMN-associated cancer is similar to PDAC [35]. The typical age for the detection of IPMNs is between the fifth and seventh decade of life and there is no difference in their prevalence between men and women [36]. IPMN diagnostics include the exclusion of the other PCLs, determination of the potential communication between lesion and MPD, and identification of the key risk factor of malignancy with assessment of the resectability. The most common location of IPMN is the pancreatic head, and multiple mural nodules, pancreatic duct dilatation, ductal communication, cyst or cluster of cysts may be imaged [36]. Tubular or earthworm-like shadows in low-density cystic lesions, cystic walls and septate microenhancing nodules in CT are specific findings of imaging for branch-type IPMNs. In contrast, main-duct IPMNs are radiologically characterized by the dilatation of the MPD, continuous expansion of the pancreatic duct without bead-like changes, enhanced mural nodules of the cyst wall, slight atrophy of the pancreatic parenchyma, and significantly dilated MPD asymmetry with mildly atrophied pancreatic parenchyma [36]. Nevertheless, the current imaging techniques possibilities in IPMNs evaluation for malignancy are very limited. Therefore, new methods and markers characterized by high sensitivity and specificity are still the subject of ongoing research. For example, Permuth and co-researchers revealed that combining radiomic features with a microRNA (miRNA) genomic classifier may improve prediction of the malignant pathology for IPMNs [37]. In fact, EUS-FNA is commonly used for IPMN diagnosis, nonetheless, an interpretation of its results should be correlated with clinical and radiological features. The aspirated fluid may be used in cytological, chemical (the level of glucose and other markers) and molecular assay. The sensitivities and specificities of biochemical analysis differ between data obtained from various research groups [38]. A pooled analysis revealed that the amylase levels lower than 250 U/l had a 98% specificity to exclude the diagnosis of pseudocyst [39]. None of the other tumor markers, such as CA 19-9, CA 125, CA 72-4, and CA 15-3, appear accurate enough to provide a definitive diagnosis [40]. The presence of significant quantities of thick mucin in the correct clinical setting may only support a diagnosis of IPMN. In addition, a differentiation of low-grade IPMN and high-dysplasia IPMN is not easy. Nevertheless, features in cytospin preparations of EUS-FNA specimen, such as necrosis, hypochromasia/hyperchromasia, the presence of large vacuole single cells, irregularities in nuclear levels, and elevated nuclear/plasmic ratio suggest the lesion with high dysplasia. Moreover, high-grade atypia of IPMN is predominantly accompanied with the cyst of a size equal or larger than 30 mm, enhanced mural nodules or solid contents and dilatation of MPD [41,42,43,44]. It is worth emphasizing that any solid component associated with a cystic lesion or regional lymph nodes may also be aspirated for cytological or histological assay [38]. IPMNs are indolent tumors characterized by favourable prognosis after surgical resection in terms of their relatively high overall survival and disease-free survival rate. Subjects with malignant pathological diagnoses should accept strict tumor surveillance after surgery in view of their higher risk of recurrence. It is important to underline that malignant IPMN detected in an early phase is characterized by the relatively good prognosis in contrast to the IPMN detected as a malignant, invasive lesion. Moreover, it is possible for an independent PDAC to coexist with benign IPMN. In this situation, the prognosis is poor. The presence of an oncocytic type of IPMN, peripheral invasion and incisal margin invasion are independent, proven factors predisposing to the recurrence [45]. It must be emphasized that there are other tools helpful in the diagnosis and management of IPMN. The so-called endoscopic "fish eye" ampulla visualized as a patulous duodenal papilla with the extrusion of mucus at duodenoscopy is typical for main duct-IPMN and combined type-IPMN [46]. Digital pancreatoscopy is a method using a peroral digital single-operator pancreatoscopy system with two light-emitting diode lights on the catheter and a single complementary metal-oxide semiconductor chip that provides improved image resolution compared to the conventional pancreatoscopy. It should be considered in the diagnostic algorithm of IPMN localized in MPD in the patients with a diffusely dilated MPD and without any focal lesions seen on imaging and EUS [47]. In turn, per-oral cholangiopancreatoscopy may be useful for pre-operative assessment of the extent of IPMN localized in MPD [48].

MCNs are the lesions originated from the pancreatic epithelium with a distinctive ovarian-type stroma having the potential for malignant transformation. The detailed malignant transformation risk of MCNs is estimated between 6% and 36% [49]. Interestingly, MCNs are found almost exclusively in women, and a female to male ratio is about 20 to 1 [49]. They are relatively infrequent PCLs and account for 29% of all PCNs [50]. In the comparison to IPMNs, MCNs do not have the connection with the pancreatic duct system and they are mostly large cysts with thick septa, peripheral calcification and mural nodules [36]. Their prevalence in the body/tail of the pancreas is higher than in the pancreatic head [51]. Irregular or focal thickening of the cyst wall, the size (>8.5 cm), a large volume, and solid content inside or outside the MCNs on CT/MR belong to the main predictors of high-grade dysplasia or invasive cancer disease. Their average growth rate is very slow (approx. 4 mm/year) [52,53,54]. In MCN, similarly to IPMN, the cystic fluid is typically characterized by high density with cytology confirming the presence of mucinous cells. It is worth emphasizing that the levels of CEA may be used to differentiate between mucinous and non-mucinous lesions [55]. CEA level of >192 μg/L is the cut-off value for the cyst differentiation with the sensitivity and specificity of 52–78% and 63–91%, respectively [56,57]. In turn, the concentration of amylase is usually normal, however a high level does not exclude MCN [31]. Nevertheless, an identification of the presence of ovarian stroma in the histological assessment is key for the diagnosis of MCNs [49].

#### 3.1.3. N-MPCLs

In this subchapter, we focus on SCNs and SPNs. SCNs are benign lesions of the pancreatic exocrine glands and account for 16–33.3% of whole PCNs. Furthermore, these changes are characterized by very low risk of malignant transformation [58,59]. SCNs occur in patients in their late 50s and early 60s, usually developing in the body or tail of the pancreas. Interestingly, despite their benign nature, they slowly grow and can reach large diameters [60]. On the other hand, SCNs are representatively honeycombed microcystic tumors consisting of uniform, cuboidal, glycogen-rich epithelial cells. Thus far, there are four variants of serous cystadenoma, namely, macrocystic serous cystadenoma, solid serous adenoma, von Hippel–Lindau-related SCN, and mixed serous neuroendocrine neoplasm, in which the serous epithelial components are identical to those of serous cystadenoma [36]. It is known that CT is the preferable method as the first-line examination for SCNs. CT scans may reveal typical features such as a star-shaped central scar with calcification, or microcystic multiple small cyst, sometimes oligocytic. The diameter of a single capsule is usually smaller than 2 cm. In turn, EUS with FNA for SCNs is characterized with low specificity and sensitivity. Due to the vascularized fibrous septa of the SCN, biopsy may be complicated with hemorrhages. In addition, the cystic fluid level of CEA was found to be low (typical <5 ng/mL) [36].

SPNs are rare tumors of the pancreas, and their frequency is assessed as only about 0.2–2.7% of all exocrine pancreatic neoplasms [61]. The origin of these tumor cells within the pancreas is uncertain. They may derive from pluripotent pancreatic cells or have genital bud origin [62]. The potential association with genital bud causes SPNs to mainly occur in young women, especially between the second and third decade of life. Regarding the typical radiological appearance of these lesions, local capsule interruption, cystic degeneration, calcification and hemorrhage, and floating cloud sign belong to the imaging features. In turn, the cytological assessment usually reveals branching papillae with myxoid stroma [36].

Despite the above-described typical features of lesions, the diagnosis may be difficult. Nevertheless, the EUS with FNA is the most accurate procedure for identification of the PCLs nature. It results from the fact that it combines cytology and a determination of the intracystic level of CEA and other biomarkers. This is important to distinguish initially mucinous from non-mucinous PCNs, because the latter have a lower predisposition to malignancy. Therefore, more accurate methods are still the subject of ongoing research. Bertani and co-researchers proved that EUS needle-based confocal endomicroscopy (nCLE) is better for the diagnosis of indeterminate PCLs compared to the EUS-FNA [63]. nCLE is a subtype of confocal laser imaging technique enabling visualization of the mucosal layer to a micron resolution [63]. The assessment of the glucose concentration in the cystic fluid became an easy and helpful biomarker. Simon-Linares and colleagues evidenced that intra-cyst glucose levels (≤41 mg/dL) may have an advantage over classic CEA testing for the differentiation of MPCLs from n-MPCLs. It was shown to be the very valuable diagnostic test with an AUC of 0.95 (95% CI: 0.81, 0.97). It must be emphasized that the intracystic levels of glucose are significantly lower in MPCLs compared to n-MPCLs [64]. The most recent data indicate that the combination of these two markers may be more effective in the diagnosis of PCLs. The co-analysis of the cyst fluid CEA and glucose at the novel cut-off values of 135.1 ng/mL and 2.8 mmol/L have the best testing performance to MPCLs [65]. Interestingly, the evaluation of individual serum tumor markers, such as CEA, CA125, and CA724, has low ability to predict advanced cystic mucinous neoplasms, and CA 19-9 represent moderate efficacy. Their combination could, perhaps, be useful, however, further studies are necessary to confirm this possibility [66]. Nevertheless, the level of CEA in the cyst fluid may be a helpful marker in differentiating mucinous from non-mucinous, but not malignant from benign PCLs. Moreover, a combined CEA and CA-125 approach may help segregate MCNs from IPMNs [67]. It is worth emphasizing that an evaluation of the cystic fluid level of kynurenine, playing an important role in the pancreatic cancer and immune biology, has been presented to have potential clinical usefulness differentiating mucinous from non-mucinous PCLs, because mucinous PCLs were found to have reduced the kynurenine level compared to non-mucinous PCLs. In addition, it may facilitate the diagnosis of serous cystadenomas that are characterized by a significant kynurenine abundance compared to lesions which are not serous cystadenomas [68].

A systematic review and meta-analysis performed by McCarty et al. demonstrated that molecular analysis of EUS-acquired pancreatic cyst fluid for KRAS and GNAS mutations was characterized by high sensitivity and specificity with significantly elevated diagnostic accuracy for diagnosis of IPMNs and MPCLs compared to CEA alone [69]. Ren and co-workers revealed that mucinous pancreatic cysts may have BRAF mutation rather than KRAS mutation, which may be used in the assessment of targeted next generation sequencing of cell-free DNA in the EUS guided workup [70]. Furthermore, marker identification of the grade of dysplasia of IPMNs in pancreatic cyst fluid using quantitative proteomic profiling has been performed, and 19 candidates with consistently increased or decreased expression correlated with IPMN malignancy features [71]. Another valuable marker may be the mucin-associated enhanced expression of MUC1, which was identified in malignant mucinous pancreatic cysts [72]. In case of low levels of CEA, some data indicate that measurements of chromogranin A (CgA) and neuron-specific enolase (NSE) in the cystic fluid may be helpful in the diagnosis of cystic neuroendocrine neoplasms [73]. Another helpful method in the diagnosis of PCLs could be ELISA with a novel monoclonal antibody Das-1 having specific immunoreactivity in tissue and cyst fluid derived from high-risk IPMN and IPMN-associated invasive carcinoma. This test has been proven to detect malignancy risk in PCLs with high levels of sensitivity (88%) and specificity (99%) [74]. Currently, an identification of the nature of Das-1 antigen and its expression pattern remains under investigation, however it is known that it occurs in a lot of organs of various origin (ectodermal, mesodermal and endodermal). Interestingly, the expression of Das-1 is present in fetal pancreatic tissue and absent in normal adult pancreas [75].

CT-based radiomics is an emerging and rapidly developing method for advanced image analysis and comprises feature extraction of various phases (plain scan, arterial phase, vein phase) of contrast-enhanced CT. Next, this action is used to construct a combination normogram incorporating a multi-phase radiomics model, involving the extraction of all three phases of CT to discriminate non-invasive subtypes of PCN. Gao and co-workers revealed that radiomics for arterial and venous single-phase models outperformed the plain scan model, and a combination nomogram that incorporated the MP-Radscore, tumor location, and cystic number had the best discriminatory performance and demonstrated excellent accuracy for differentiating SCN from MCN [76]. In addition, multi-phase post-CT radiomic evaluation could improve predictive capability in diagnosis of the malignancy in IPMNs and has benefits over single venous phase CT analysis [77]. Furthermore, a combination of cross-sectional imaging features and blood markers that can readily be obtained by non-invasive examination during the surveillance period, may distinguish benign from malignant IPMNs. Multivariable logistic regression analysis demonstrated that cyst size, cyst location, cyst wall enhancement, multicystic lesion, diameter of MPD, neutrophil-to-lymphocyte ratio, serum CA 19-9, and CEA were significantly associated with high risk of malignancy. The normogram, constructed based on these variables, revealed excellent discrimination power with an AUC of 0.859 (95% CI: 0.818–0.900, *p* < 0.001) [78].

### 3.2. Solid Lesions

Solid lesions are the main cause of pancreatectomy among PIs [79]. They have high malignant potential, and the differential diagnosis, especially in incidentally detected small solid lesions, can be difficult [2,80].

#### 3.2.1. PDAC

PDAC is the most common cause of the solid PIs [12]. It is highly lethal, but the early diagnosis is challenging. Most of the patients with symptomatic PDAC have advanced-stage diseases. Incidental PDAC is mainly detected during other pathologies’ diagnostics or new-onset/exacerbation of diabetes mellitus evaluation. Incidentally diagnosed PDAC is characterized by smaller tumor size, higher resectability and better prognosis compared to the symptomatic PDAC [81]. The transabdominal US reveals PDAC as an irregular hypoechoic mass related to an abrupt cut-off of the MPD and upstream MPD dilatation, and sensitivity of this imaging method for the diagnosis of PDAC ranges from 75–89%, depending on numerous factors including, among others, the experience of the operator and the presence of bowel gases [82,83]. PDAC usually has a dense fibroblastic stroma and, therefore, typically appears as a hypoattenuating mass compared to normal pancreatic parenchyma during the pancreatic phase of CT, whereas MR demonstrates PDAC as hypointense to normal pancreatic change on fat-suppressed T1-weighted images and hypointense to isointense on post-contrast T1-weighted images [84].

Data from Japan showed that 40% of asymptomatic PDAC cases were already in stage III or IV, and the 5-year overall survival was 30.6%, which is far below the average 5-year overall survival for all cancer types (64.1%) [81]. Interestingly, despite better prognosis of asymptomatic PDAC (earlier diagnosis), screening for PDAC is not actually recommended in the general population and it results from the lack of non-invasive, useful biomarkers at the early stages of disease, relatively low prevalence of PDAC and the patients’ distress associated with screening tests [85,86]. Interestingly, it is estimated that approximately 60% of the patients with incidentally diagnosed PDAC were characterized by an elevation of CA 19-9 level [81]. On the other hand, CA 19-9 could be an anchor marker for application of the pancreatic cancer early detection. In the cohorts of the prostate, lung, colorectal and ovarian cancer screening trial, CA 19-9 levels exponentially raised starting at 2 years before the PDAC diagnosis with sensitivities reaching 60% and 99% specificity within 6 months before PDAC diagnosis, and 50% at 99% specificity for early PDAC. Some authors suggest that CA 19-9 may be routinely evaluated in patients at high risk of PDAC, however, this observation should be confirmed by further studies [87,88]. It is important to remember that the value of the elevated level of CA 19-9 in the early detection of PDAC is not fully satisfactory. Its sensibility is insufficient, and its specificity is rather low, resulting in expensive, unnecessary diagnostic workup in the majority of cases. For example, Illés et al. (2016) tried to determine the incidence of PDAC in new-onset type 2 diabetic patients by measuring the serum level of CA 19-9 in group of 115 patients. The normal level of CA 19-9 was in two of three detected cancer cases (not measured in the third one). In addition, PDAC was not found in 10 patients who had abnormally increased level of CA 19-9 [89]. Therefore, the measurement of CA 19-9 level should be used with caution during early detection of PDAC.

The detection of PDAC as PI is not enough to ensure long-term survival. Ideally, PDAC should be diagnosed when cancer is still not detectable using the current imaging techniques or when it is not clinically evident. For example, abnormal dilatation of the MPD with the absence of visible lesion may represent the image of the early-staged PDAC [84,90]. Interestingly, if a patient with suspected PDAC does not have dilatation of MPD, and the fine-needle aspiration is negative for malignancy, the likelihood of cancer is low [91]. Furthermore, focal parenchymal atrophy of the pancreas on CT may serve as an important imaging sign for the early diagnosis of PDAC [92,93]. In addition, there is evidence demonstrating that focal parenchymal atrophy might be the earliest sign of PDAC, which appears before MPD abnormalities [92]. Novel biomarkers, such as non-coding RNAs, exosomes, and circulating DNA, have been reported to improve the accuracy of diagnosis and could contribute to the facilitation of the incidental PDAC diagnosis [94,95,96]. However, intensive studies are still in progress to identify both diagnostic, predictive and prognostic markers that would facilitate the diagnosis of PDAC detected incidentally [97].

#### 3.2.2. pNET

pNET is second leading cause of the solid PIs. Incidentally diagnosed pNETs are usually characterized as being smaller than 2 cm and with a G1 differentiation grade. Furthermore, in the comparison to the symptomatic pNETs, pNETs as PIs have higher rate of pancreatic-sparing resections (62% vs. 30%), but there was no change in the rate of perioperative morbidity and mortality: 5-year disease-free survival was higher for incidental pNET (92% vs. 82%) [98]. Solid pNETs are generally radiologically characterized by intense enhancement on the arterial phase with traditional cross-sectional imaging, and therefore are well-differentiated from PDAC [99]. pNETs may be divided into functioning and non-functioning. Non-functioning pNETs produce nonspecific peptides, such as chromogranin A, but also may secrete low quantity of hormones, most commonly pancreatic polypeptide and calcitonin [100]. Non-functioning pNETs are more dominant as PIs than functioning pNETs [5]. The majority of pNETs are detected in the body and tail of the pancreas and are often accompanied by arterial enhancement. The minority of pNETs (about 18%) contain calcifications. It is worth emphasizing that pNETs may have cystic structure, however, it is relatively rare [101]. Between 50 and 90% of non-functioning pNETs are malignant, but unlike ductal adenocarcinomas, they have a more indolent character and better prognosis [102]. Characteristics of solid pNETs, such as size >3 cm, duct dilatation, vascular invasion, enlargement of peripancreatic lymph nodes, and calcification, suggest malignant character. Interestingly, a cut-off sizes of 2 and 3 cm result in a positive prospective value for malignancy of 44% and 61%, respectively [103]. Data indicate that intrapancreatic accessory spleen and serous cystadenoma should be primarily taken into account in the differential diagnosis of solid pNETs [104]. In the diagnostic process of pNETs, including their metastases, octreotide scintigraphy, single-photon emission computed tomography (SPECT) and positron emission tomography (PET)/CT with radioactively labelled somatostatin analogs should be considered and may be helpful when results of CT and MR are doubtful. It results from the fact that 50–90% of pNETs express the somatostatin receptors [105,106]. As mentioned above, it is important to determine whether a lesion is functioning. Therefore, biochemical diagnosis of pNETs is required. It is suggested that evaluation of the serum levels of chromogranin A, pancreatic polypeptide, glucose, insulin, gastrin, and glucagon could detect functional pNETs in asymptomatic patients. Both the North American (NANETS) and European (ENETS) Neuroendocrine Tumor Societies recommend measurement of chromogranin A in both non-functioning and functioning tumors, as well as evaluation of specific hormones, such as insulin, C-peptide, pro-insulin, gastrin, glucagon, vasoactive intestinal peptide, parathyroid hormone-related protein, adrenocorticotropic hormone, and somatostatin based on clinical manifestations [5]. However, recommendations according to small, asymptomatic, incidental pNETs are not clear. In the patients’ group, a measurement of chromogranin A, as well as pancreatic polypeptide and calcitonin, are recommended. Importantly, a thorough medical history is required to rule out multiple endocrine neoplasia (MEN) type 1. Currently, studies evaluating novel markers of pNETs are still in progress [107]. Nevertheless, there are some markers that deserve special attention. Circulating tumor cells (CTCs), the tumor cells in the peripheral blood, may be useful biomarkers for providing diagnostic and prognostic information, their presence is associated with higher tumor grade, tumor burden, an increased circulating chromogranin A concentration and higher Ki67 index [108]. Another novel biomarker of pNETs is a novel multianalyte biomarker, multiple transcript analysis PCR-based test (NETest) using blood-based quantitative real-time PCR to measure 51 different NET-related transcripts presented promising results in both the diagnosis and prognosis of the disease [109]. This method may be used in prognosis and response to treatment in follow-up. In addition, it seems that NETest is more informative than an evaluation of changes of chromogranin A in predicting alterations of the disease [110,111]. Moreover, microRNA seems to be valuable marker, and downregulation of serum microRNA-1290 may serve as discriminator pNET from PDAC [112].

Poorly differentiated PNETs generally present with metastases and are not generally subjected to resection. Well- or intermediately differentiated tumors ≥2 cm with imaging evidence of malignancy or with a Ki-67 >2% should be resected. It has been suggested that non-MEN related, non-functioning, and asymptomatic PNETs <2 cm with a Ki-67 index ≤2% carry a low risk of metastasis and may be observed in the absence of clinical or radiologic criteria of malignancy or progression, especially in older patients [5].

#### 3.2.3. Solid Pseudopapillary Tumor

Solid pseudopapillary tumor belongs to the rare lesions of the pancreas, and its frequency is estimated to be between 0.9–2.7% of all exocrine pancreatic neoplasms in adults. Most of them are asymptomatic and incidentally detected [113]. A single-institution study performed by Kotecha and co-researchers revealed that 91% of patients taking part in this research were female, indicating the potential predisposition of the female sex to this type of pancreatic lesion [114]. Moreover, it primarily occurs in the third and fourth decade of life [115]. It is worth emphasizing that rare solid pseudopapillary tumor may be associated with familial adenomatous polyposis [116,117]. The tail of pancreas is described as the most common location in adults, whereas the pancreatic head is the main locality in children [118,119]. The potential of malignancy of solid pseudopapillary tumor is low, which contributes to good prognosis, and aggressive biological behaviour of the lesion characterized by the recurrence and distant metastasis occurs occasionally [120]. Generally, solid pseudopapillary tumors are round, well-demarcated and large, and their mean size ranges from 8 to 10 cm [113]. Microscopically, they are composed of both solid and pseudopapillary structures with areas of necrosis and hemorrhages [115]. It must be emphasized that solid pseudopapillary tumor and pNET have a close resemblance on imaging and cytomorphology, though they differ in their prognosis and treatment strategy. Cytomorphology of samples collected from EUS-FNA or surgical resection is crucial in differentiating SPN from pNET, and nuclear folds have vital significance for distinguishing these pathologies (nuclear fold grades two and three are more representative for solid pseudopapillary tumor than pNET, similar to metachromatic matrix, hyaline globules, and nuclear grooves typical for solid pseudopapillary tumor) [121]. Smears are generally richly cellular, and their backgrounds consist of multinucleated giant cells, foamy macrophages, and hemorrhagic debris. Characteristic myxoid clear material surrounding the papillae, the existence of cercariform cells and of foamy histiocytes or multinucleated giant cells are additional significant cytological features [122,123,124]. Nevertheless, immunohistochemistry is mandatory for the final diagnosis, and adequate choice of diagnostic antibodies is key. β-catenin and CD99 (dotlike pattern) as positive markers, and chromogranin, trypsin, BCL10 and E-cadherin as negative markers, are recommended for immunohistochemical panel used to the diagnosis of solid pseudopapillary tumor [113].

#### 3.2.4. Focal Chronic Pancreatitis and Autoimmune Pancreatitis

Generalized parenchymal glandular atrophy, diffuse pancreatic calcifications, and irregular dilatation of the MPD are the most frequent imaging findings of chronic pancreatitis. Hypovascular mass with calcifications, irregularity of MPD, and duct penetrating sign likely correspond to focal chronic pancreatitis. In addition, pseudocysts may occasionally occur [125]. It is worth emphasizing that differentiating between pancreatic cancer and focal chronic pancreatitis, usually located in the pancreas head, is difficult and results from overlapping features [126]. Longer and more gradually tapered stenosis of the common bile duct at the level of the pancreatic head is observed in focal chronic pancreatitis compared to PDAC, and may be accompanied with the abdominal pain and recurrent acute pancreatitis episodes [127]. In addition, the assessment of duct/parenchyma ratio and superior mesentric artery/superior mesentric vein ratio in CT may facilitate the diagnosis of incidentally diagnosed focal chronic pancreatitis and distinguishing from pancreatic cancer [91,128,129,130].

Fibrosis of the pancreas occurs in both chronic pancreatitis and PDAC. Nevertheless, the detailed evaluation of pancreatic fibrosis may facilitate diagnosis and differentiation with other diseases, in which fibrotic changes occur. A diffusion of fibrotic component unaccompanied with the demarcation of the pancreatic mass indicate chronic pancreatitis. Moreover, it is worth emphasizing that chronic pancreatitis may lead to PDAC and result from one that causes chronic obstruction of the MPD distal to the mass lesion. Therefore, it is important to suspect and exclude cancer in the patients with incidentally detected focal chronic pancreatitis. Nevertheless, oligosymptomatic chronic pancreatitis of long evolution is not rare, but in general the morphological alterations are diffuse. The current imaging techniques are not efficient in detecting PDAC in the course of chronic pancreatitis [131,132]. The coexistence of calcifications displaced by the mass, any abnormal contour bulge, and/or change in the form of mass effect double-duct sign, vascular invasion, and pancreatic cut-off may suggest pancreatic cancer [133,134].

Autoimmune pancreatitis is a distinct form of pancreatitis that can be associated with IgG4 laden lymphoplasmacytic infiltration and fibrosis in multiple organs. Painless jaundice, mild abdominal pain, recurrent acute pancreatitis and response to steroids or other immunomodulatory therapy are characteristic of the disease. There are two types of autoimmune pancreatitis. Patients with type 1 disease usually demonstrate painless obstructive jaundice, affect mainly men over 60 years, which is often accompanied by extrapancreatic organs involvement. In turn, type 2 autoimmune pancreatitis is characterized by younger age of the disease onset, more frequent presentation with acute pancreatitis, and the absence of extrapancreatic involvement [135]. Interestingly, autoimmune pancreatitis may be detected incidentally [136]. It is important to remember that the “icicle” sign (the dilated upstream MPD proximal to the site of involvement in focal type of autoimmune pancreatitis) or “ice pick” sign (a smooth tapered narrowing of the upstream MPD distal to the pancreatic lesion) can simplify the detailed diagnosis of PIs and indicate chronic pancreatitis, because they are frequently seen in autoimmune pancreatitis [137].

#### 3.2.5. Others

Mesenchymal tumors of the pancreas belong to rare tumors and account for 1–2% of all pancreatic tumors. Among these, lipoma and liposarcoma, fat-originating lesions, are the rarest and may be incidentally detected [138,139]. To emphasize the fact that these lesions may be PIs despite low incidence and enhancement of knowledge, we focus on them in this part of the review.

A lipoma is an encapsulated mass consisting of mature adipose cells arranged in lobules. In addition, it can contain fine connective tissue septa. A thin capsule differentiates lipoma from lipomatosis, that is, focal fat replacement as deposition of the fatty tissue in the pancreatic parenchyma in continuity with the peripancreatic fat [139,140]. The majority of lipomas are located in the head of pancreas and are less than 5 cm in size, however giant tumors have also been reported [138,139,141]. In the diagnosis of lipoma, ultrasound is not the reference method, because this tumor may be hypo-, iso- or hyperechoic lesion. For this reason, CT is recommended and demonstrates lipoma as a homogeneously hypodense, non-enhancing mass with density values ranging from 30 to 120 Hounsfield units with distinct margins and no infiltration of surrounding tissues. In turn, MR presents lipoma as hyperintensive lesion on both T1 and T2-weighted sequences, similar to intra-abdominal and subcutaneous fat [138,139,140]. Histological diagnostics are used in only a few cases, usually after surgical intervention [142].

In turn, liposarcoma is itself a heterogeneous group, and its classification is divided into subtypes based on morphologic features and cytogenetic aberrations, namely well-differentiated, de-differentiated, myxoid, round cell and pleomorphic [143]. The well-differentiated and myxoid/round cell are forms with slow growth, low metastatic potential and progressive destruction of normal pancreatic tissue. In contrast, the undifferentiated and pleomorphic type are lesions with high-grade malignancy, remarkable biological aggressiveness and higher metastatic potential [144,145]. It is worth emphasizing that liposarcoma of the pancreas is very rare, and occurs more frequently in limbs and retroperitoneum [146]. In opposition to lipoma, the liposarcoma is characterized by poorly defined areas of higher density on CT images. Moreover, it seems that MR is the preferred method of imaging that enables potential diagnosis of the type of liposarcoma based on an assessment of the signal intensify on T1- and T2-weighted images [144]. Male sex predominance, the presence of thick septations and internal calcifications within the tumor are characteristics differentiating liposarcoma from lipoma [140]. Generally, surgical resection is currently the only potentially curative therapy for the liposarcoma [146].

### 3.3. Abnormal Dilatation of the MPD

There is no consensus regarding the diameter size criteria that defines pancreatic duct dilatation, but a diameter of greater than 3 mm in the head, and greater than 2 mm upstream to the head is often considered dilated. The lack of precise limit values results from the fact that there are variations of the MPD related to age [125,147]. Therefore, it is very important to determine features that could indicate malignant cause of the dilatation of incidentally detected MPD. Kim et al. (2017) revealed that patients with benign MPD dilatation, transition areas were frequently located in the head (57.9% vs. 13.6%, *p* = 0.003) and demonstrated significantly shorter (<6.1 mm) (78.9% vs. 9.1%, *p* < 0.0001) and smooth transition (89.5% vs. 9.1%, *p* < 0.0001) compared to the malignant MPD dilatation. Duct penetrating sign, defined as a thin intact PD visible traversing the transition zone in CT, was exclusively observed in patients with benign MPD dilatation (73.7% vs. 0%, *p* < 0.0001). In contrast, malignant MPD dilatation was frequently characterized by attenuation difference (63.6% vs. 10.5%, *p* = 0.001) and associated common bile duct enhancement (36.4% vs. 0%, *p* = 0.003) in comparison to subjects with benign MPD dilatation [148]. In turn, Fujisawa et al. proved that marked pancreatic duct dilatation (≥3.5 mm) and elevated HbA1c (≥6.1%) strongly suggest the presence of pancreatic diseases among other pancreatic cyst or chronic pancreatitis. In addition, if these features are accompanied by obesity, PDAC should be ruled out [13]. In conclusion, the incidentally diagnosed dilatation of MPD should be carefully assessed. In absence of CP, an MD/mixed-type IPMN should be suspected. It needs to be remembered that the presence of CP does not exclude the existence of IPMN [149]. Recently, pancreatoscopy is proposed to investigate MPD and facilitate differentiating between benign causes and neoplasm related to the dilatation of MPD [150].

## 4. Management

**Figure 2:**
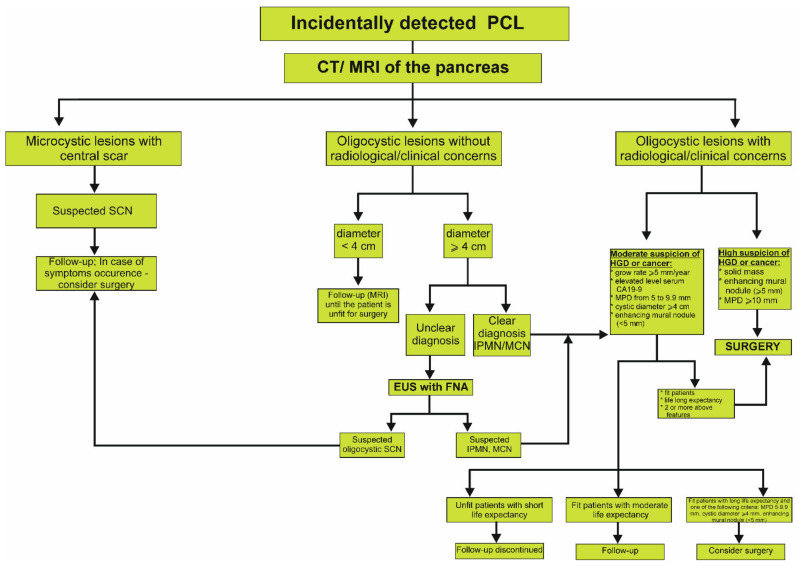
Proposed algorithm of management of incidentally detected cystic lesion in the pancreas based on the European Evidence-Based Guidelines on Cystic Tumors of the Pancreas [2,56]. CT, computed tomography; EUS, endoscopic ultrasound; FNA, fine needle aspiration; HGD, high grade dysplasia; IPMN, intraductal papillary mucinous neoplasm; MCN, mucinous cystic neoplasm; MPD, main pancreatic duct; MRI, magnetic resonance imaging; PCL, pancreatic cystic lesion; SCN, serous cystic neoplasm.

**Figure 3:**
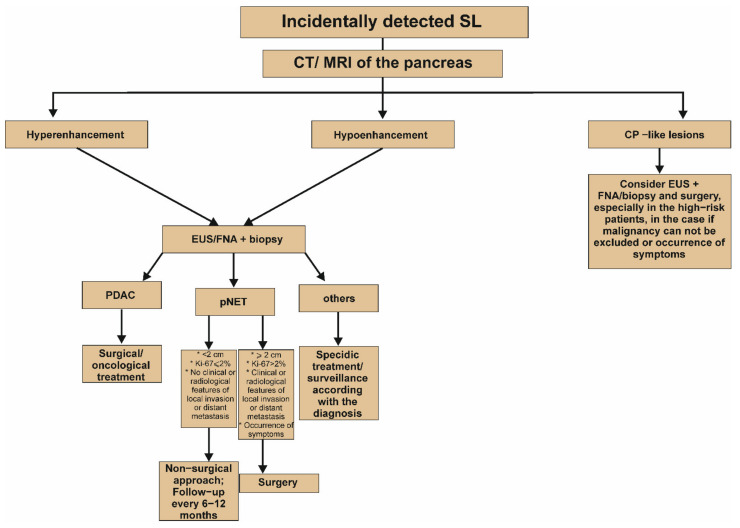
Proposed algorithm of management of incidentally detected solid lesion in the pancreas [2]. It is worth emphasizing that the Figure presents a simplified algorithm. It should be noted that differentiation between hypo- and hyperenhancing change is significant at the initial stages of the diagnostic process and may strongly determine further clinical management. For example, hypoenhancing lesion concomitant with upstream dilatation of the main pancreatic duct could be a clear indication for surgery, even without EUS with or without FNA. In turn, surgery should be also considered in the case of hypoenhancing lesion, whose fine needle aspiration biopsy does not exclude the cancer diagnosis. CP, chronic pancreatitis; CT, computed tomography; EUS, endoscopic ultrasound; FNA, fine needle aspiration; Ki-67, antigen KI-67; MRI, magnetic resonance imaging; PDAC, pancreatic ductal adenocarcinoma; pNET, pancreatic neuroendocrine tumor; SL, solid lesion.

**Figure 4:**
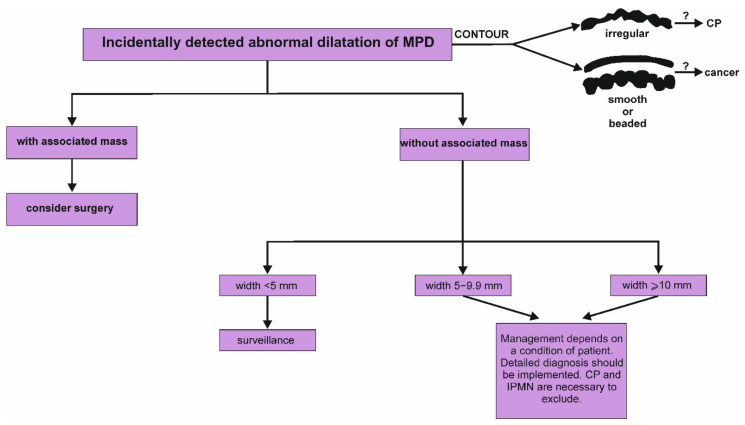
Proposed algorithm of management of incidentally detected dilatation of main pancreatic duct in the pancreas [2]. It is worth emphasizing that the assessment of the contours of main pancreatic duct may be significant and potentially indicate a cause of dilatation. Irregular contour with a dilatation of MPD suggests the presence of periductal fibrosis and is crucial sign of chronic pancreatitis. In turn, smooth or beaded dilatation of the main pancreatic duct may result from pancreatic cancer. CP, chronic pancreatitis; IPMN, intraductal papillary mucinous neoplasm; MPD, main pancreatic duct.

Because of the serious overlap in the morphology of benign and premalignant lesions, characterizing and managing PIs poses a significant problem for the clinicians. All of the described groups of PIs have worrisome characteristics that should be included for making adequate therapeutic decision. Schemes demonstrating the management of PIs with cystic lesion, solid lesion and the MPD dilatation are represented in Figure 2, Figure 3 and Figure 4, respectively. Nevertheless, the clinical condition of patients is the most important issue in making decisions about surgery treatment, and it is key to remember age, life expectancy, health status, degree of frailty, patient preference, motivation for surgery, and availability of benefit. Each patient should be carefully evaluated by clinicians, including a surgeon and gastroenterologist, according to the patient’s own situation after adequate consultation. Another significant factor in the decision is the surgery type, as pancreaticoduodenectomy or distal pancreatectomy have different responsibilities in terms of mortality. There are two aspects that should guide the management of PIs: whether the PIs is malignant and whether the PIs will become malignant during a patient’s lifetime. In the second situation, regular control diagnosis is of key importance. For example, based on the guidelines of the American Gastroenterological Association Institute, the patients with pancreatic cysts <3 cm without a solid component or a dilated MPD should undergo MRI after 1 year. If there is no change in size or characteristics, they should undergo MRI every 2 years afterward for a total of 5 years [151]. It is worth emphasizing that cytological examination is usually the first and most used method in the diagnostic workup of cystic and solid PIs, and based on the analysis of the different markers in the cystic fluid aspirate the diagnosis and prognosis may be assumed [113]. Nevertheless, EUS and the aspiration of cyst fluid are not performed in the majority of patients with the unequivocal image in the everyday clinical practice. The adequate diagnostic approach and knowledge about characteristic individual types of PIs may facilitate proper diagnosis and the implementation of appropriate management. In the case of non-malignant change, it may result in the avoidance of unneeded surgical operation, as well as overtreatment and unnecessary anxiety of patients.

## 5. Conclusions

In recent years, the incidence of PIs has increased. The choice of appropriate management has key significance and is dependent on the characteristics of the lesion. The detection of PIs may be not associated with the necessity of immediate implementation of invasive procedures. Nonetheless, the management of PI should begin with a dedicated pancreas protocol CT or MRI to characterize the MPD size, lesion characteristics and establish an accurate baseline for subsequent follow-up. Newer, cheaper, more accurate diagnostic methods and protocols, including distinguishing between benign and premalignant lesions, as well as the development of novel prognostic and diagnostic biomarkers characterized by high efficacy and utility are needed. It could facilitate diagnosis and save stress for the patients.

## Abbreviations

CP	chronic pancreatitis
IPMN	intraductal papillary mucinous neoplasm
PDAC	pancreatic ductal adenocarcinoma
PI	pancreatic incidentaloma
pNET	pancreatic neuroendocrine tumor
